# First-line pembrolizumab ± chemotherapy for recurrent/metastatic head and neck cancer: Japanese subgroup of KEYNOTE-048

**DOI:** 10.1007/s10147-022-02233-6

**Published:** 2022-10-20

**Authors:** Shunji Takahashi, Nobuhiko Oridate, Kaoru Tanaka, Yasushi Shimizu, Yasushi Fujimoto, Koji Matsumoto, Tomoya Yokota, Tomoko Yamazaki, Masanobu Takahashi, Tsutomu Ueda, Nobuhiro Hanai, Hironori Yamaguchi, Hiroki Hara, Tomokazu Yoshizaki, Ryuji Yasumatsu, Masahiro Nakayama, Kiyoto Shiga, Takashi Fujii, Kenji Mitsugi, Kenichi Takahashi, Nijiro Nohata, Burak Gumuscu, Ramona F. Swaby, Makato Tahara

**Affiliations:** 1 Cancer Institute Hospital, Japanese Foundation For Cancer Research, 3-8-31 Ariake, Koto-ku, Tokyo, 135-8500 Japan; 2 Yokohama City University Graduate School of Medicine, 4-57 Urafune, Minami-ku, Yokohama, 236-0004 Japan; 3 Kindai University Hospital, 377-2 Ohno-Higashi, Osaka-Sayama, 589-8511 Japan; 4 Hokkaido University Hospital, 5 Chome Kita 14 Jonishi, Kita Ward, Sapporo, Hokkaido 060-8648 Japan; 5 Aichi Medical University Hospital, Yazako, Karimata-1-1, Nagakute, Aichi 480-1195 Japan; 6 Hyogo Cancer Center, 1370 Akashi, Hyogo, 673-0021 Japan; 7 Shizuoka Cancer Center, 1007 Shimonagakubo, Nagaizumi, Sunto District, Shizuoka, 411-8777 Japan; 8 Miyagi Cancer Center, 47-1 Nodayama Medeshimashiote, Natori, Miyagi 981-1293 Japan; 9 Tohoku University Hospital, 1-1 Seiryomachi, Aoba-ku, Sendai, Miyagi 980-8574 Japan; 10 Hiroshima University Hospital, 1 Chome-2-3 Kasumi, Minami Ward, Hiroshima, 734-8551 Japan; 11 Aichi Cancer Center, Yazako, Karimata-1-1, Nagakute, Aichi 480-1195 Japan; 12 Jichi Medical University, 3311-1 Yakushiji, Shimotsuke, Tochigi 329-0498 Japan; 13 Saitama Cancer Center, 780 Komuro, Ina, Kitaadachi District, Saitama, 362-0806 Japan; 14 Kanazawa University, Kakumamachi, Kanazawa, Ishikawa 920-1192 Japan; 15 Kyushu University, 744 Motooka, Nishi-ku, Fukuoka, 819-0935 Japan; 16 Tsukuba University, 1 Chome-1-1 Tennodai, Tsukuba, Ibaraki 305-8577 Japan; 17 Iwate Medical University, 19-1 Uchimaru, Morioka, Iwate 020-0023 Japan; 18 Osaka International Cancer Institute, 1-3-3 Nakamichi, Tosei-ku, Osaka, 537-8511 Japan; 19 Hamanomachi Hospital, 3-chōme-3-1 Nagahama, Chuo Ward, Fukuoka, 810-8539 Japan; 20 MSD K.K., Kitanomaru Square, 1-chōme-13-12 Kudankita, Chiyoda City, Tokyo 102-0073 Japan; 21 Merck & Co., Inc., 126 E Lincoln Ave, Rahway, NJ 07065 USA; 22 Present Address: CARISMA Therapeutics Inc., Philadelphia, PA USA; 23 National Cancer Center Hospital East, 6-5-1 Kashiwanoha, Kashiwa, Chiba 277-8577 Japan

**Keywords:** Head and neck squamous cell carcinoma, Pembrolizumab, First-line treatment, Combined positive score, PD-L1, Japan

## Abstract

**Background:**

Here, we report the results of the Japanese subgroup of the phase 3 KEYNOTE-048 study of pembrolizumab alone, pembrolizumab plus platinum and 5-fluorouracil (pembrolizumab–chemotherapy), or cetuximab plus platinum and 5-fluorouracil (EXTREME) in previously untreated recurrent/metastatic (R/M) head and neck squamous cell carcinoma (HNSCC).

**Methods:**

Primary end points were overall survival (OS) and progression-free survival (PFS). Efficacy was evaluated in patients with PD-L1 combined positive score (CPS) ≥ 20 and ≥ 1 and the total Japanese subgroup (*n* = 67).

**Results:**

At data cutoff (25 February 2019), pembrolizumab led to longer OS versus EXTREME in the PD-L1 CPS ≥ 20 subgroup (median, 28.2 vs. 13.3 months; HR, 0.29 [95% CI 0.09–0.89]) and to similar OS in the total Japanese (23.4 vs. 13.6 months; HR, 0.51 [95% CI 0.25–1.05]) and CPS ≥ 1 subgroups (22.6 vs. 15.8 months; HR, 0.66 [95% CI 0.31–1.41]). Pembrolizumab–chemotherapy led to similar OS versus EXTREME in the PD-L1 CPS ≥ 20 (median, 18.1 vs. 15.8 months; HR, 0.72 [95% CI 0.23–2.19]), CPS ≥ 1 (12.6 vs. 15.8 months; HR, 1.19 [95% CI 0.55–2.58]), and total Japanese subgroups (12.6 vs. 13.3 months; unadjusted HR, 1.10 [95% CI 0.55–2.22]). Median PFS was similar for pembrolizumab and pembrolizumab–chemotherapy versus EXTREME in all subgroups. Grades 3–5 treatment-related adverse events occurred in 5 (22%), 19 (76%), and 17 (89%) patients receiving pembrolizumab, pembrolizumab–chemotherapy, and EXTREME, respectively. One patient receiving pembrolizumab–chemotherapy died because of treatment-related pneumonitis.

**Conclusion:**

These results support the use of first-line pembrolizumab and pembrolizumab–chemotherapy for Japanese patients with R/M HNSCC.

*Clinical trial registry* ClinicalTrials.gov, NCT02358031.

**Supplementary Information:**

The online version contains supplementary material available at 10.1007/s10147-022-02233-6.

## Introduction

Head and neck squamous cell carcinomas (HNSCC) are anatomically heterogenous, often aggressive malignancies commonly associated with tobacco use, alcohol consumption, and human papillomavirus (HPV) infection [1, 2]. The incidence of head and neck cancers varies significantly by country and are particularly common in Japan [3, 4]. The incidence of lip, oral cavity, and pharynx cancers in Japanese men in 2014 was 21.6 per 100,000 population compared with a world incidence of 9.4 per 100,000, and the incidence of larynx cancer was 7.8 per 100,000 compared with 2.9 per 100,000, respectively [4]. Although the incidence is lower in women, it is still significantly more common in Japanese women than in the global population (lip, oral cavity, pharynx cancer: 8.4 per 100,000 vs. 3.2 per 100,000; larynx cancer: 0.5 per 100,000 vs. 0.2 per 100,000).

Despite improvements in management and diagnostics, more than 65% of patients with HNSCC develop recurrent or metastatic (R/M) disease, which has a poor prognosis [2]. Until recently, the standard of care for R/M disease in the USA and Japan was cetuximab with a platinum-based agent and 5-fluorouracil—the EXTREME regimen [5–8]. However, lately, immune checkpoint inhibitors as first- and second-line treatments have demonstrated significant survival benefits in HNSCC [9–11].

KEYNOTE-048 was a phase 3 study evaluating pembrolizumab alone and in combination with chemotherapy in previously untreated R/M HNSCC [11]. In KEYNOTE-048, pembrolizumab monotherapy significantly prolonged overall survival (OS) in patients with a PD-L1 combined positive score (CPS) of ≥ 20 and CPS ≥ 1 and had noninferior OS in the total population compared with EXTREME. Safety of pembrolizumab was favorable compared with EXTREME. Pembrolizumab plus platinum and 5-fluorouracil (pembrolizumab–chemotherapy) significantly prolonged OS in patients with PD-L1 CPS ≥ 20, CPS ≥ 1, and in the total population compared with EXTREME. Safety of pembrolizumab–chemotherapy was comparable with that of EXTREME. Based on these results, pembrolizumab is approved in Japan as a first-line treatment option as monotherapy and in combination with platinum and 5-fluorouracil for all patients with R/M HNSCC, regardless of PD-L1 CPS [12].

Given the high incidence of HNSCC in Japan [4], it is important to investigate the efficacy and safety of pembrolizumab in patients of Japanese ethnicity. Here, we report the final analysis of the Japanese subgroup of KEYNOTE-048.

## Materials and methods

### Study design and patients

KEYNOTE-048 was a randomized, phase 3 study in previously untreated R/M HNSCC (Clinicaltrials.gov, NCT02358031). The methods have been reported previously [11]. Briefly, patients were adults with histologically or cytologically confirmed R/M SCC of the oropharynx, oral cavity, hypopharynx, or larynx incurable by local therapy; had an Eastern Cooperative Oncology Group (ECOG) performance status (PS) of 0/1; measurable disease per Response Evaluation Criteria in Solid Tumours, version 1.1 (RECIST v1.1); and known p16 expression for oropharyngeal cancers (nonoropharyngeal cancers were considered HPV negative). Patients were stratified by percentage of tumor cells expressing PD-L1 (≥ 50% vs. < 50%), HPV status for oropharyngeal cancers (p16 positive vs. negative), and ECOG PS (0 vs. 1) and randomized 1:1:1 to pembrolizumab monotherapy, pembrolizumab plus platinum and 5-fluoruracil (pembrolizumab–chemotherapy), or cetuximab plus platinum and 5-flurouracil (EXTREME). Only patients enrolled in Japan were included in this analysis.

The study protocol and all amendments were conducted in accordance with Good Clinical Practice Guidelines and approved by the appropriate ethics committee at each center. All patients provided written informed consent.

### Procedures

In the pembrolizumab monotherapy and pembrolizumab–chemotherapy arms, pembrolizumab (200 mg) was administered once every 3 weeks (Q3W). Chemotherapy in the pembrolizumab–chemotherapy and EXTREME arms comprised carboplatin (area under the curve 5 mg/m^2^) or cisplatin (100 mg/m^2^) and 5-fluorouracil (1000 mg/m^2^ per day for 4 consecutive days) Q3W for 6 cycles. Patients in the EXTREME arm also received cetuximab (400-mg/m^2^ loading dose, then 250 mg/m^2^ per week). Study treatment continued until ≤ 35 administrations of pembrolizumab, disease progression, intolerable toxicity, or withdrawal.

Imaging was performed at baseline, week 9, and then Q6W until year 1, and Q9W thereafter. Response assessments used RECIST v1.1, with confirmation by blinded independent central review. Safety was assessed throughout the study and for 30 days after completion (90 days for serious adverse events [AEs]). AEs were graded per the National Cancer Institute Common Terminology Criteria for Adverse Events, version 4.0.

### Outcomes

Primary end points were OS and progression-free survival (PFS). Secondary end points included safety and tolerability, PFS rates at 6 and 12 months, and objective response rate (ORR). Duration of response (DOR) was an exploratory endpoint. Efficacy was evaluated in patients with PD-L1 CPS ≥ 20, with PD-L1 CPS ≥ 1, and in the total Japanese subgroup.

### Statistical analyses

Efficacy was assessed in the intention-to-treat (ITT) population of all patients randomly allocated to treatment. Safety was assessed in all patients who received ≥ 1 dose of study treatment. OS, PFS, and DOR were estimated using the Kaplan–Meier method. Additional methods on the Cox proportional hazards model are provided in the Supplement.

## Results

Of the 882 patients enrolled in the KEYNOTE-048 study, 67 were enrolled in Japan (pembrolizumab monotherapy, *n* = 23; pembrolizumab–chemotherapy, *n* = 25; EXTREME, *n* = 19). Baseline disease characteristics were generally similar between treatment arms (Supplementary Table 1), although there were differences in the proportion of patients with an ECOG PS of 0 (pembrolizumab monotherapy vs. EXTREME, 74% vs. 42%; pembrolizumab–chemotherapy vs. EXTREME, 48% vs. 44%), with a PD-L1 tumor proportion score of more than 50% (26% vs. 32% and 8% vs. 38%, respectively), who were current or former smokers (87% vs. 95% and 84% vs. 94%), and who had metastatic disease (87% vs. 89% and 64% vs. 81%).

All patients received ≥ 1 dose of study drug (Supplementary Fig. 1). At data cutoff (25 February 2019), 22 (96%) patients in the pembrolizumab arm, 25 (100%) in the pembrolizumab–chemotherapy arm, and 18 (95%) in the EXTREME arm discontinued treatment, mainly because of progressive disease. Subsequent anticancer therapies were received by 16 (70%), 11 (44%), and 14 (74%) patients in the pembrolizumab, pembrolizumab–chemotherapy, and EXTREME arms, respectively (Supplementary Table 2). Median follow-up was 36.8 months (range 26.3–44.2 months) for pembrolizumab monotherapy versus 35.1 months (range 25.3–44.4 months) for EXTREME and 34.3 months (range 25.7–45.7 months) for pembrolizumab–chemotherapy versus 34.2 months (range 25.3–44.4 months) for EXTREME (Supplementary Table 3).

### Efficacy

**Fig. 1:**
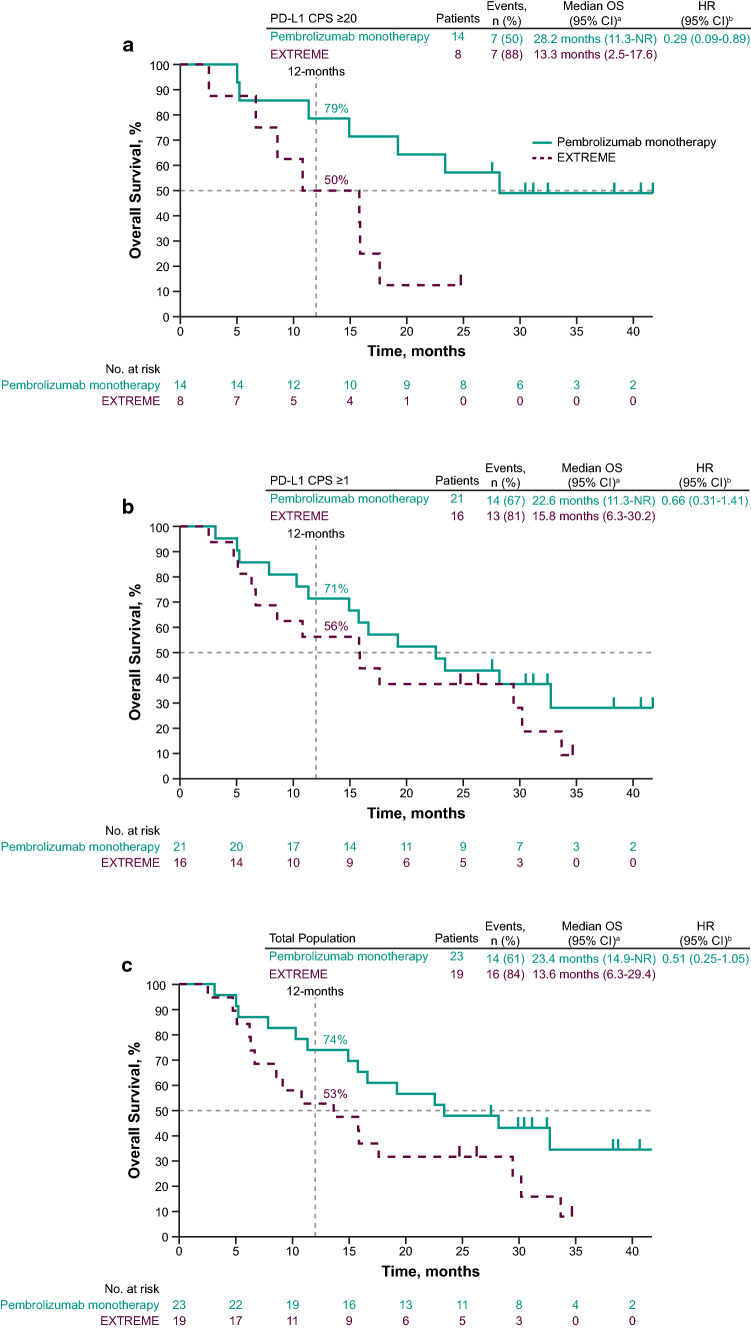
Kaplan–Meier estimates of overall survival in the Japanese subgroup. Pembrolizumab monotherapy versus EXTREME in the **a** PD-L1 CPS ≥ 20, **b** PD-L1 CPS ≥ 1, and **c** total Japanese subgroups. ^a^From product-limit (Kaplan–Meier) method for censored data. ^b^Based on Cox regression model with Efron’s method of tie handling, with treatment as a covariate. CI, confidence interval; CPS, combined positive score; EXTREME, cetuximab plus platinum and 5-fluorouracil; HR, hazard ratio; NR, not reached; OS, overall survival; PD-L1, programmed death ligand 1

Median OS for pembrolizumab monotherapy versus EXTREME in the PD-L1 CPS ≥ 20 subgroup was 28.2 months (95% CI 11.3 months–not reached [NR]) versus 13.3 months (95% CI 2.5–17.6 months) (HR, 0.29; 95% CI 0.09–0.89) (Fig. 1A). In the PD-L1 CPS ≥ 1 subgroup, the median OS was 22.6 months (95% CI 11.3 months–NR) for pembrolizumab monotherapy versus 15.8 months (95% CI 6.3–30.2 months) for EXTREME (HR, 0.66; 95% CI 0.31–1.41) (Fig. 1B). The median OS in the total Japanese subgroup was 23.4 months (95% CI 14.9 months–NR) for pembrolizumab monotherapy versus 13.6 months (95% CI 6.3–29.4 months) for EXTREME (HR, 0.51; 95% CI 0.25–1.05) (Fig. 1C). The 12-month OS rates were 79% versus 50%, 71% versus 56%, and 74% versus 53% for pembrolizumab monotherapy versus EXTREME in the PD-L1 CPS ≥ 20, PD-L1 CPS ≥ 1, and total Japanese subgroups, respectively.

**Fig. 2:**
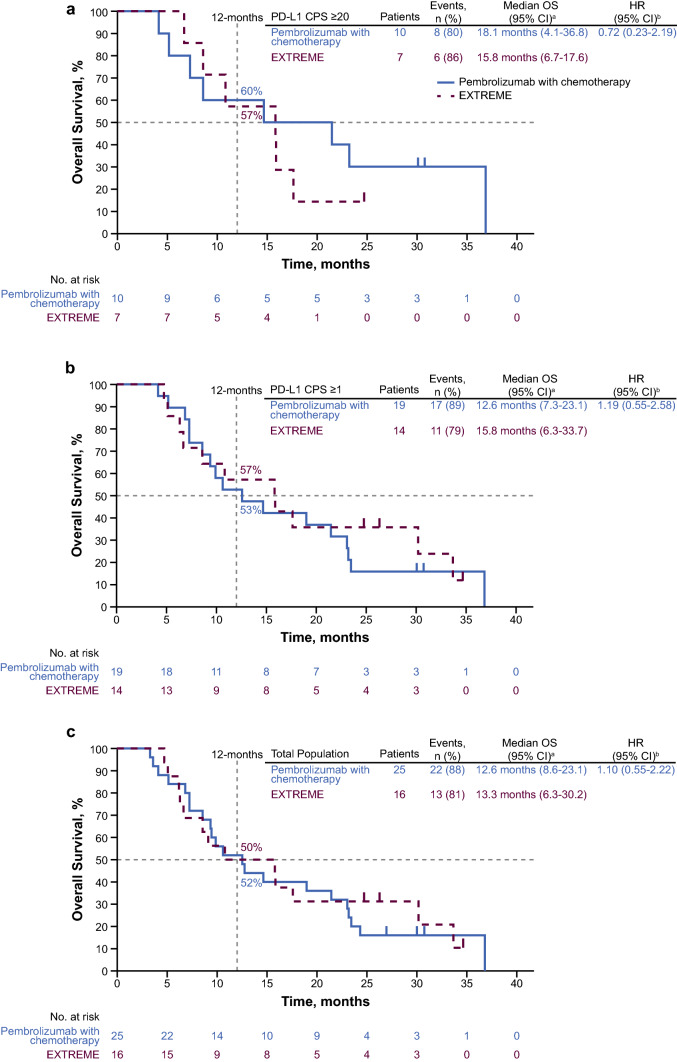
Kaplan–Meier estimates of overall survival in the Japanese subgroup. Pembrolizumab–chemotherapy versus EXTREME in the **a** PD-L1 CPS ≥ 20, **b** PD-L1 CPS ≥ 1, and **c** total Japanese subgroups. ^a^From product-limit (Kaplan–Meier) method for censored data. ^b^Based on Cox regression model with Efron’s method of tie handling, with treatment as a covariate. CI, confidence interval; CPS, combined positive score; EXTREME, cetuximab plus platinum and 5-fluorouracil; HR, hazard ratio; NR, not reached; OS, overall survival; PD-L1, programmed death ligand 1

Median OS for pembrolizumab–chemotherapy versus EXTREME in the PD-L1 CPS ≥ 20 subgroup was 18.1 months (95% CI 4.1–36.8 months) versus 15.8 months (95% CI 6.7–17.6 months) (HR, 0.72; 95% CI 0.23–2.19) (Fig. 2A). In the PD-L1 CPS ≥ 1 subgroup, the median OS was 12.6 months (95% CI 7.3–23.1 months) for pembrolizumab–chemotherapy versus 15.8 months (95% CI 6.3–33.7 months) for EXTREME (HR, 1.19; 95% CI 0.55–2.58) (Fig. 2B). The median OS in the total Japanese subgroup was 12.6 months (95% CI 8.6–23.1 months) for pembrolizumab–chemotherapy versus 13.3 months (95% CI 6.3–30.2 months) for EXTREME (unadjusted HR, 1.10; 95% CI 0.55–2.22; adjusted HR, 0.88; 95% CI 0.41–1.88) (Fig. 2C). The 12-month OS rates were 60% versus 57%, 53% versus 57%, and 52% versus 50% for pembrolizumab–chemotherapy versus EXTREME in the PD-L1 CPS ≥ 20, PD-L1 CPS ≥ 1, and total Japanese subgroups, respectively.

**Fig. 3:**
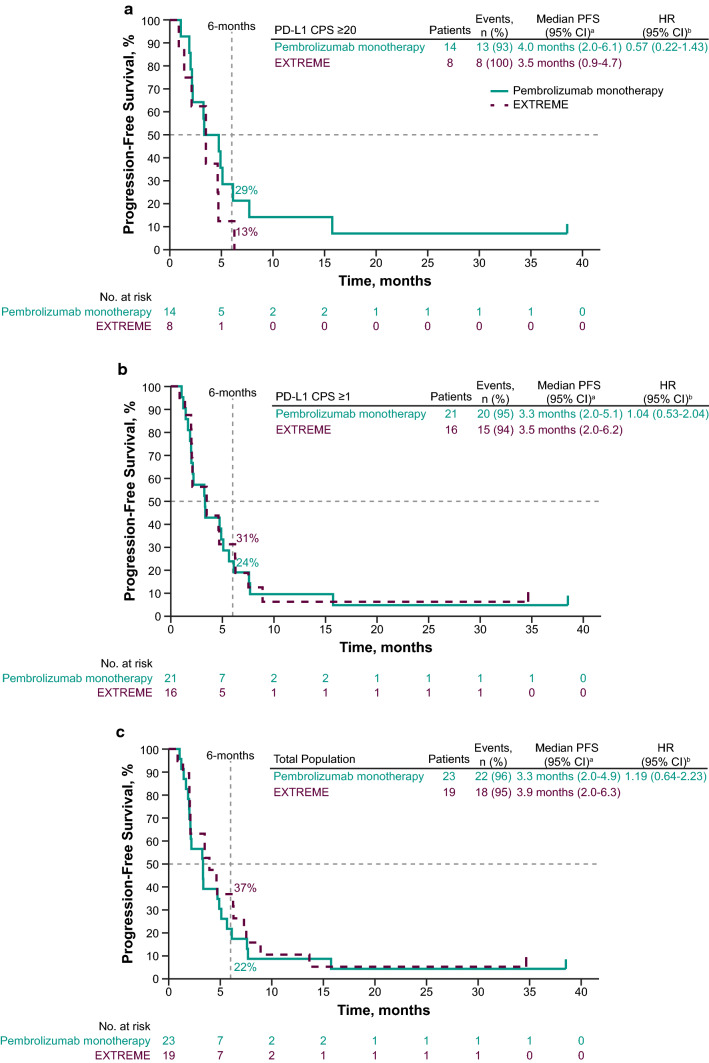
Kaplan–Meier estimates of progression-free survival in the Japanese subgroup. Pembrolizumab monotherapy versus EXTREME in the **a** PD-L1 CPS ≥ 20, **b** PD-L1 CPS ≥ 1, and **c** total Japanese subgroups. ^a^From product-limit (Kaplan–Meier) method for censored data. ^b^Based on Cox regression model with Efron’s method of tie handling, with treatment as a covariate. CI, confidence interval; CPS, combined positive score; EXTREME, cetuximab plus platinum and 5-fluorouracil; HR, hazard ratio; NR, not reached; PD-L1, programmed death ligand 1; PFS, progression-free survival

Median PFS for pembrolizumab versus EXTREME in the PD-L1 CPS ≥ 20 subgroup was 4.0 months (95% CI 2.0–6.1 months) versus 3.5 months (95% CI 0.9–4.7 months) (HR, 0.57; 95% CI 0.22–1.43) (Fig. 3A). In the PD-L1 CPS ≥ 1 subgroup, the median PFS was 3.3 months (95% CI 2.0–5.1 months) for pembrolizumab monotherapy versus 3.5 months (95% CI 2.0–6.2 months) for EXTREME (HR, 1.04; 95% CI 0.53–2.04 months) (Fig. 3B). The median PFS in the total Japanese subgroup was 3.3 months (95% CI 2.0–4.9 months) for pembrolizumab monotherapy versus 3.9 months (95% CI 2.0–6.3 months) for EXTREME (HR, 1.19; 95% CI 0.64–2.23) (Fig. 3C).

**Fig. 4:**
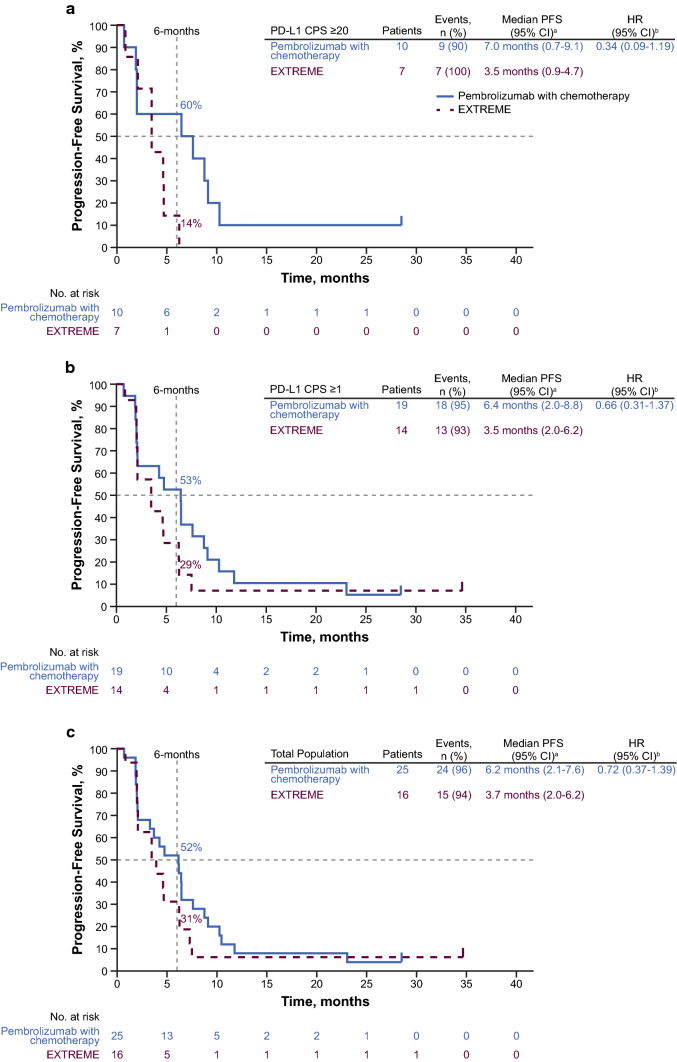
Kaplan–Meier estimates of progression-free survival in the Japanese subgroup. Pembrolizumab–chemotherapy versus EXTREME in the **a** PD-L1 CPS ≥ 20, **b** PD-L1 CPS ≥ 1, and **c** total Japanese subgroups. ^a^From product-limit (Kaplan–Meier) method for censored data. ^b^Based on Cox regression model with Efron’s method of tie handling with treatment as a covariate. CI, confidence interval; CPS, combined positive score; EXTREME, cetuximab plus platinum and 5-fluorouracil; HR, hazard ratio; NR, not reached; PD-L1, programmed death ligand 1; PFS, progression-free survival

Median PFS for pembrolizumab–chemotherapy versus EXTREME in the PD-L1 CPS ≥ 20 subgroup was 7.0 months (95% CI 0.7–9.1 months) versus 3.5 months (95% CI 0.9–4.7 months) (HR, 0.34; 95% CI 0.09–1.19) (Fig. 4A). In the PD-L1 CPS ≥ 1 subgroup, the median PFS was 6.4 months (95% CI 2.0–8.8 months) for pembrolizumab–chemotherapy versus 3.5 months (95% CI 2.0–6.2 months) for EXTREME (HR, 0.66; 95% CI 0.31–1.37) (Fig. 4B). The median PFS in the total Japanese subgroup was 6.2 months (95% CI 2.1–7.6 months) for pembrolizumab–chemotherapy versus 3.7 months (95% CI 2.0–6.2 months) for EXTREME (HR, 0.72; 95% CI 0.37–1.39) (Fig. 4C).

The ORR for pembrolizumab monotherapy versus EXTREME in the PD-L1 CPS ≥ 20 subgroup was 29% (1 complete response [CR], 3 partial responses [PRs]) versus 13% (1 PR), and the median DOR was 8.4 versus 2.6 months (Supplementary Table 4). In the PD-L1 CPS ≥ 1 subgroup, the ORR was 19% (1 CR, 3 PRs) for pembrolizumab monotherapy versus 25% (1 CR, 3 PRs) in the EXTREME arm, and the median DOR was 8.4 versus 5.5 months. In the total Japanese subgroup, the ORR was 17% (1 CR, 3 PRs) for pembrolizumab monotherapy versus 37% (1 CR, 6 PRs) for EXTREME; the median DOR was 8.4 versus 4.1 months.

The ORR for pembrolizumab–chemotherapy versus EXTREME in the PD-L1 CPS ≥ 20 subgroup was 50% (1 CR, 4 PRs) versus 14% (1 PR), and the median DOR was 6.9 versus 2.6 months (Supplementary Table 5). In the PD-L1 CPS ≥ 1 subgroup, the ORR was 32% (1 CR, 5 PRs) for pembrolizumab–chemotherapy versus 21% for EXTREME (1 CR, 2 PRs); the median DOR was 7.5 versus 4.1 months. In the total Japanese subgroup, the ORR was 32% (1 CR, 7 PRs) for pembrolizumab–chemotherapy and 31% (1 CR, 4 PRs) for EXTREME; the median DOR was 7.5 versus 4.1 months.

**Fig. 5:**
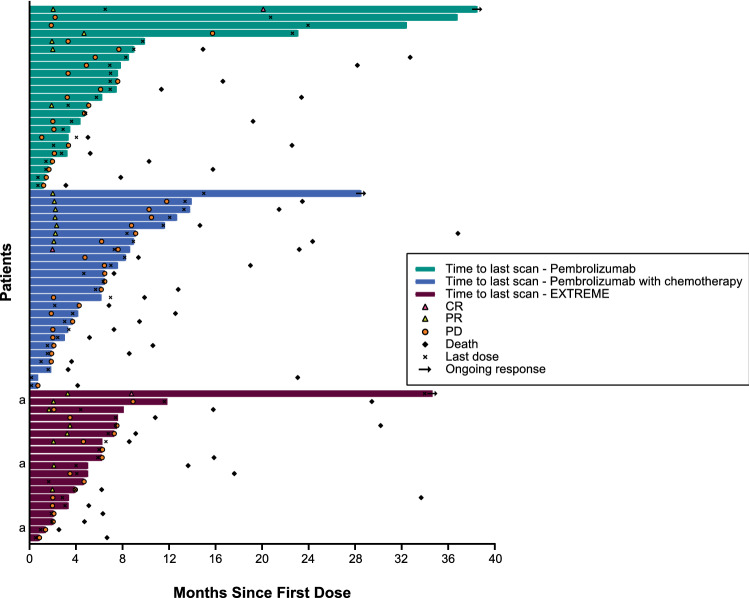
Treatment exposure and response duration in the Japanese subgroup receiving pembrolizumab monotherapy, pembrolizumab–chemotherapy, and EXTREME. ^a^Patients in the EXTREME arm who were not included in the comparison analysis of pembrolizumab–chemotherapy versus EXTREME because they were enrolled in the EXTREME arm while enrollment in the pembrolizumab–chemotherapy arm was temporarily halted. CR, complete response; EXTREME, cetuximab plus platinum and 5-fluorouracil; PD, progressive disease; PR, partial response

**Fig. 6:**
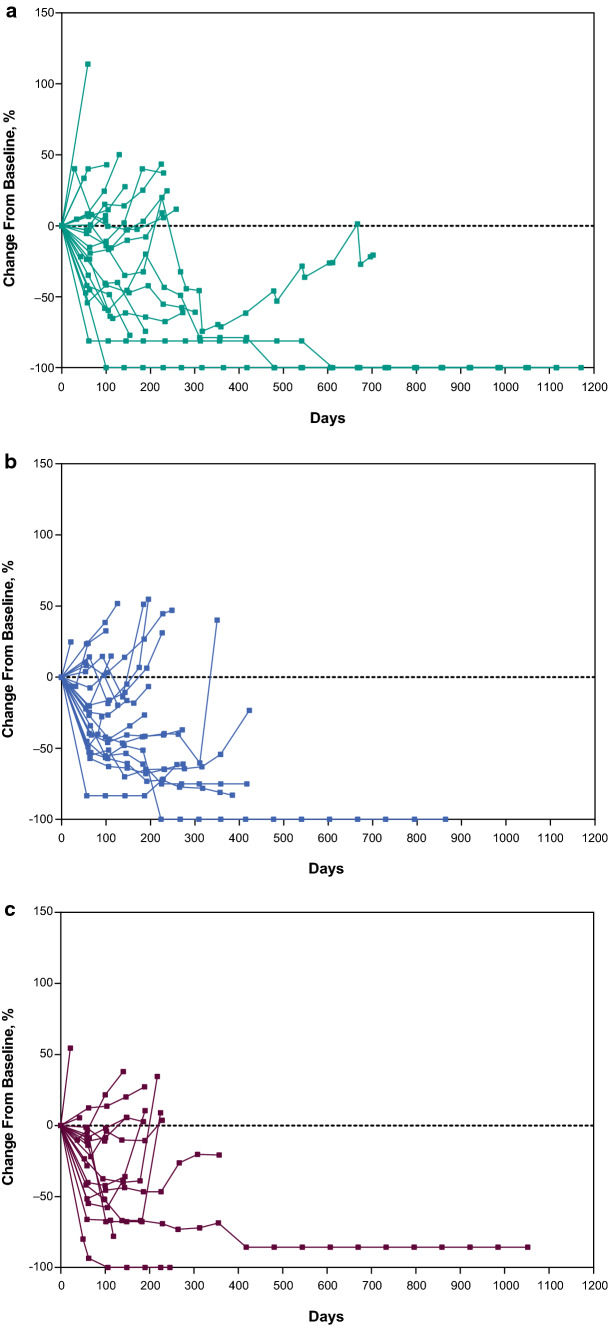
Change from baseline in target lesion size in the Japanese Subgroup receiving **a** pembrolizumab monotherapy (*n* = 23), **b** pembrolizumab with chemotherapy (*n* = 24),^a^ and **c** EXTREME (*n* = 19). ^a^Target lesion size data with confirmation by blinded independent central review were not available for one patient in the pembrolizumab–chemotherapy arm. EXTREME, cetuximab plus platinum and 5-fluorouracil

Responses were durable with pembrolizumab monotherapy and pembrolizumab–chemotherapy (Fig. 5, Supplementary Tables 4, 5). Response was ongoing for 2 patients with CR (1, pembrolizumab monotherapy; 1, EXTREME), and 1 with PR (pembrolizumab–chemotherapy) (Fig. 5). Analysis of change from baseline in target lesion size showed reductions were generally durable over time for pembrolizumab monotherapy and pembrolizumab–chemotherapy (Fig. 6A–C).

### Safety

Any-grade treatment-related AEs (TRAEs) occurred in 17 (74%), 25 (100%), and 19 (100%) patients in the pembrolizumab monotherapy, pembrolizumab–chemotherapy, and EXTREME arms, respectively (Supplementary Table 6). Grade 3–5 TRAEs occurred in 22%, 76%, and 89% of patients in the pembrolizumab monotherapy, pembrolizumab–chemotherapy, and EXTREME arms, respectively. No grade 3–5 TRAEs occurred in > 1 patient receiving pembrolizumab monotherapy. The most common grade 3–5 TRAEs in the pembrolizumab–chemotherapy arm were neutrophil count decreased (40%), white blood cell (WBC) count decreased (36%), anemia (32%); and in the EXTREME arm, neutrophil count decreased (58%), WBC count decreased (47%), and anemia (32%). Serious TRAEs occurred in 3 (13%), 7 (28%), and 5 (26%) patients in the pembrolizumab monotherapy, pembrolizumab–chemotherapy, and EXTREME arms, respectively. TRAEs led to discontinuation of any drug in 2 (9%), 1 (4%), and 3 (16%) patients in the pembrolizumab monotherapy, pembrolizumab–chemotherapy, and EXTREME arms, respectively. One patient receiving pembrolizumab–chemotherapy died because of treatment-related pneumonitis.

Immune-mediated AEs (imAEs) and infusion reactions occurred in 35% of patients in the pembrolizumab monotherapy arm, 32% in the pembrolizumab–chemotherapy arm, and 21% in the EXTREME arm (Table 1). The most common was hypothyroidism, which occurred in 9%, 16%, and 11% of patients in the pembrolizumab monotherapy, pembrolizumab–chemotherapy, and EXTREME arms, respectively; all were grade 1/2. Grade 3–5 imAEs included grade 4 hepatitis (4%) and grade 3 hypophysitis (4%), nephritis (4%), and severe skin reaction (9%) in the pembrolizumab monotherapy arm, grade 5 pneumonitis (4%) and grade 3 severe skin reaction (4%) in the pembrolizumab–chemotherapy arm, and grade 3 infusion reaction (5%) in the EXTREME arm.

**Table 1:** Summary of immune-mediated adverse events and infusion reactions that occurred in ≥ 1 patient in any treatment arm in the Japanese subgroup

	Pembrolizumab monotherapy *n* = 23				Pembrolizumab with chemotherapy *n* = 25				EXTREME *n* = 19			
	Grade 1/2	Grade 3	Grade 4	Grade 5	Grade 1/2	Grade 3	Grade 4	Grade 5	Grade 1/2	Grade 3	Grade 4	Grade 5
Any	4 (17)	3 (13)	1 (4)	0	6 (24)	1 (4)	0	1 (4)	3 (16)	1 (5)	0	0
Hypothyroidism	2 (9)	0	0	0	4 (16)	0	0	0	2 (11)	0	0	0
Infusion reaction	1 (4)	0	0	0	1 (4)	0	0	0	1 (5)	1 (5)	0	0
Pneumonitis	1 (4)	0	0	0	0	0	0	1 (4)	1 (5)	0	0	0
Severe skin reaction	0	2 (9)	0	0	0	1 (4)	0	0	0	0	0	0
Colitis	0	0	0	0	1 (4)	0	0	0	0	0	0	0
Hepatitis	0	0	1 (4)	0	0	0	0	0	0	0	0	0
Hypophysitis	0	1 (4)	0	0	0	0	0	0	0	0	0	0
Hyperthyroidism	0	0	0	0	1 (4)	0	0	0	0	0	0	0
Nephritis	0	1 (4)	0	0	0	0	0	0	0	0	0	0

## Discussion

These results from the Japanese subgroup analysis of previously untreated R/M HNSCC in KEYNOTE-048 were generally consistent with those of the global population, particularly with respect to pembrolizumab monotherapy [11]. In the global KEYNOTE-048 population, pembrolizumab monotherapy significantly prolonged OS in the PD-L1 CPS ≥ 20 and CPS ≥ 1 populations and had noninferior OS in the total population versus EXTREME, and pembrolizumab–chemotherapy significantly prolonged OS in all populations. No improvement in PFS or ORR was observed in either pembrolizumab arm. In the current analysis, pembrolizumab monotherapy showed an OS benefit versus EXTREME in the PD-L1 CPS ≥ 20 subgroup, and similar OS in the total Japanese and PD-L1 CPS ≥ 1 subgroups. PFS was similar between the pembrolizumab monotherapy and EXTREME arms. OS and PFS were similar between pembrolizumab–chemotherapy and EXTREME in all subgroups. Responses were durable with both pembrolizumab and pembrolizumab–chemotherapy, as expected, given the known ability of anti-PD-1 therapies to produce durable responses [13].

Because this post hoc analysis was based on a small subgroup of patients, it was not powered to show differences in efficacy for pembrolizumab monotherapy or pembrolizumab–chemotherapy versus EXTREME. The current analysis is limited by the low number of patients enrolled in Japan, and consequently the small sizes of the PD-L1 CPS subgroups, which resulted in wide 95% confidence intervals for survival estimates. In addition, differences in baseline characteristics between treatment arms may have influenced the results. More patients in the pembrolizumab monotherapy arm versus the EXTREME arm had an ECOG PS of 0 (74% vs. 42%), whereas the proportion was similar in the pembrolizumab–chemotherapy and EXTREME arms (48% vs. 44%). This may have contributed to the improved survival seen in the pembrolizumab monotherapy arm in the Japanese subgroup. In contrast, ECOG PS was balanced between treatment arms in the overall study population as it was a stratification factor for randomization. The adjusted hazard ratio for OS suggested a treatment benefit for pembrolizumab–chemotherapy versus EXTREME in the total Japanese subgroup, although with a wide confidence interval.

The larger proportion of Japanese patients (61%) who received subsequent anticancer therapy versus the total population (48%) may also have impacted the results. As expected, more patients in the Japanese subgroup who received pembrolizumab monotherapy (57%) and pembrolizumab–chemotherapy (32%) received a subsequent epidermal growth factor receptor inhibitor than those in the EXTREME arm (11%), and more patients in the EXTREME arm (47%) received subsequent anti-PD-1/anti-PD-L1 therapy than those in the pembrolizumab monotherapy (9%) or pembrolizumab–chemotherapy (0%) arms. The higher proportion of patients in the EXTREME arm who received subsequent immunotherapy may have led to OS being higher than expected. Thus, the survival benefit from subsequent immunotherapy in the EXTREME arm of the Japanese subgroup may have confounded the OS results in the current analysis.

Limited data are available regarding the efficacy of targeted therapies and immunotherapies in Japanese patients with R/M HNSCC. A phase 2 study of EXTREME in Japan reported a median OS of 14.1 months, median PFS of 4.1 months, and an ORR per RECIST of 45% (*N* = 33) [14]. These results are consistent with those of the EXTREME arm in the current report (median OS, 13.6 months; median PFS, 3.9 months; ORR, 37%), noting that the sample size in both studies was relatively small. Similar results were reported from a retrospective study of first-line EXTREME in Japan, showing median OS of 11 months and a median PFS of 5 months [7]. A subanalysis of the Asia-Pacific region, including Japan, of the phase 1b KEYNOTE-012 study of pembrolizumab in R/M HNSCC reported an ORR of 19%, a median OS of 11.6 months, and a median PFS of 2.1 months [15]. Although this ORR was similar to that observed in the pembrolizumab monotherapy arm (ORR, 17%), the median OS in the current analysis was substantially longer (median OS, 23.4 months). However, only 15% of patients in the KEYNOTE-012 subanalysis had treatment-naive disease; almost half had received ≥ 3 prior lines of therapy for R/M disease, likely contributing to the limited response. In comparison, a real-world study of nivolumab in patients with R/M HNSCC reported an ORR of 21.8%, a median OS of NR, and a median PFS of 25.0 weeks [16]. Although there were differences in the patient populations—29.5% of patients had received first-line nivolumab and 16.1% had non-SCC cancers—these results are generally similar to those of the pembrolizumab monotherapy arm in the current analysis. An Asian subanalysis of the CheckMate 141 study of nivolumab has been reported; however, most patients had platinum-refractory HNSCC, and results are therefore not comparable to the current analysis [17].

The safety of pembrolizumab monotherapy and pembrolizumab–chemotherapy was similar in the Japanese subgroup and the global population [11]. Any-grade and grade 3–5 TRAEs were less frequent for pembrolizumab monotherapy compared with pembrolizumab–chemotherapy and EXTREME. The incidence of grade 3–5 TRAEs in the EXTREME arm (89%) was similar to that observed in the phase 2 study of cetuximab combined with cisplatin and 5-fluorouracil in Japanese patients with HNSCC (97%) [14]. The incidence of imAEs was similar in all treatment arms, with hypothyroidism being most common. Overall, pembrolizumab had favorable safety and pembrolizumab–chemotherapy had comparable safety versus EXTREME.

In this analysis, pembrolizumab monotherapy and pembrolizumab plus chemotherapy demonstrated efficacy and manageable safety in Japanese patients with previously untreated HNSCC. These results support the use of pembrolizumab or pembrolizumab–chemotherapy as first-line therapy for Japanese patients with R/M HNSCC.

## Supplementary Information

Below is the link to the electronic supplementary material.

Supplementary file1 (DOCX 390 KB) Methods: study design and patients, procedures, outcomes, statistical analyses. Table S1. Baseline characteristics of the Japanese subgroup. Table S2. Summary of subsequent anticancer therapy for the Japanese subgroup of KEYNOTE-048. Table S3. Time from randomization to data cutoff (25 February 2019). Table S4. Summary of confirmed objective response by PD-L1 CPS for Japanese patients receiving pembrolizumab monotherapy vs EXTREME. Table S5. Summary of confirmed objective response by PD-L1 CPS for Japanese patients receiving pembrolizumab with chemotherapy vs EXTREME. Table S6. Summary of treatment-related adverse events that occurred in ≥ 2 patients in any treatment arm of the Japanese subgroup. Figure S1. Trial population for the Japanese subgroup of KEYNOTE-048
