# Supplementary material for: First-line pembrolizumab ± chemotherapy for recurrent/metastatic head and neck cancer: Japanese subgroup of KEYNOTE-048
Source: Int J Clin Oncol. 2022 Oct 20;27(12):1805–17. doi: 10.1007/s10147-022-02233-6 (PMC9700657; doi:10.1007/s10147-022-02233-6)

**First-Line Pembrolizumab ± Chemotherapy for Recurrent/Metastatic Head and Neck Cancer: Japanese Subgroup of KEYNOTE-048**

*International Journal of Clinical Oncology*

Shunji Takahashi^1^; Nobuhiko Oridate^2^; Kaoru Tanaka^3^; Yasushi Shimizu^4^; Yasushi Fujimoto^5^; Koji Matsumoto^6^; Tomoya Yokota^7^; Tomoko Yamazaki^8^; Masanobu Takahashi^9^; Tsutomu Ueda^10^; Nobuhiro Hanai^11^; Hironori Yamaguchi^12^; Hiroki Hara^13^; Tomokazu Yoshizaki^14^; Ryuji Yasumatsu^15^; Masahiro Nakayama^16^; Kiyoto Shiga^17^; Takashi Fujii^18^; Kenji Mitsugi^19^; Kenichi Takahashi^20^; Nijiro Nohata^20^; Burak Gumuscu^21^; Ramona Swaby^21,a^; Makato Tahara^22^

^1^Cancer Institute Hospital, Japanese Foundation For Cancer Research, 3-8-31, Ariake, Koto-ku, Tokyo, 135-8500, Japan, s.takahashi-chemotherapy@jfcr.or.jp; ^2^Yokohama City University Graduate School of Medicine, 4-57 Urafune, Minami-ku, Yokohama, 236-0004, Japan, noridate@yokohama-cu.ac.jp; ^3^Kindai University, 3-4-1 Kowakae, Higashiosaka City, Osaka, 577-8502, Japan, katanaka@med.kindai.ac.jp; ^4^Hokkaido University Hospital, 5 Chome Kita 14 Jonishi, Kita Ward, Sapporo, Hokkaido, 060-8648, Japan, y-simz@med.hokudai.ac.jp; ^5^Aichi Medical University Hospital, Nagakute, Yazako, Karimata-1-1, Aichi, 480-1195, Japan, yasushif@aichi-med-u.ac.jp; ^6^Hyogo Cancer Center, 1370 Akashi, Hyogo, 673-0021, Japan, kojmatsu2@hyogo-cc.jp; ^7^Shizuoka Cancer Center, 1007 Shimonagakubo, Nagaizumi, Sunto District, Shizuoka, Japan, t.yokoya@scchr.jp; ^8^Miyagi Cancer Center, Nodayama-47-1 Medeshimashiote, Nator, Miyagi, 981-1293, Japan, tomoko-yamazaki@miyagi-pho.jp; ^9^Tohoku University Hospital, 1-1 Seiryomachi, Aoba-ku, Sendai, Miyagi, 980-8574, Japan, masanobu.takahashi.a7@tohoku.ac.jp; ^10^Hiroshima University Hospital, 1 Chome-2-3 Kasumi, Minami Ward, Hiroshima, 734-8551, Japan, uedatsu@hiroshima-u.ac.jp; ^11^Aichi Cancer Center, Nagakute, Yazako, Karimata-1-1, Aichi, 480-1195, Japan, hanai@aichi-cc.jp; ^12^Jichi Medical University, 3311-1 Yakushiji, Shimotsuke, Tochigi, 329-0498, Japan, yamaguchi@jichi.ac.jp; ^13^Saitama Cancer Center, 780 Komuro, Ina, Kitaadachi District, Saitama, 362-0806, Japan, hirhara@saitama-pho.jp; ^14^Kanazawa University, Kakumamachi, Kanazawa, Ishikawa, 920-1192, Japan, tomoy@med.kanazawa-u.ac.jp; ^15^Kyushu University, 744 Motooka, Nishi-ku, Fukuoka, 819-0935, Japan, yasuryuj@qent.med.kyushu-u.ac.jp; ^16^Tsukuba University, 1 Chome-1-1 Tennodai, Tsukuba, Ibaraki, 305-8577, Japan, nnmasa@md.tsukuba.ac.jp; ^17^Iwate Medical University, 19-1 Uchimaru, Morioka, Iwate, 020-0023, Japan, kshiga@iwate-med.ac.jp; ^18^Osaka International Cancer Institute, 1-3-3 Nakamichi, Tosei-ku, Osaka, 537-8511, Japan, takashi.fujii@oici.jp; ^19^Hamanomachi Hospital, 3-chōme-3-1 Nagahama, Chuo Ward, Fukuoka, 810-8539, Japan, mitsugi-k@hamanomachi.jp; ^20^MSD K.K., Kitanomaru Square, 1-chōme-13-12 Kudankita, Chiyoda City, Tokyo, 102-0073, Japan, kenichi.takahashi@merck.com, nijiro.nohata@merck.com; ^21^Merck & Co., Inc., 126 E Lincoln Ave, Rahway, NJ, 07065, USA, burak.gumuscu@merck.com, rfswaby@gmail.com; ^22^National Cancer Center Hospital East, 6-5-1 Kashiwanoha, Kashiwa, Chiba, 277-8577, Japan, matahara@east.ncc.go.jp.

**Corresponding author email address:**

[matahara@east.ncc.go.jp](mailto:matahara@east.ncc.go.jp)

^a^An employee of Merck Sharp & Dohme Corp., a subsidiary of Merck & Co., Inc., Kenilworth, NJ, USA at the time the study was conducted.

Supporting information of additional methods, references, supplementary tables 1-6, and supplementary figure 1 are included.

SUPPORTING INFORMATION

**Methods**

**Study design and patients**

Enrollment of patients in the pembrolizumab with chemotherapy arm was temporarily halted after 3 deaths occurred among the first 14 patients allocated to this treatment arm (August 13, 2015). Enrollment in this arm was resumed on October 2, 2015, as previously reported^1^ once the data and safety monitoring committee had reviewed safety data from 20 participants receiving pembrolizumab with chemotherapy who had completed 2 cycles of treatment.

**Procedures**

PD-L1 expression was assessed using the PD-L1 IHC 22C3 pharmDx (Agilent Technologies, Carpinteria, CA, USA) and characterized by combined positive score (CPS). CPS was defined as the number of PD-L1–staining cells (tumor cells, lymphocytes, and macrophages) divided by the total number of viable tumor cells, multiplied by 100.

**Outcomes**

Overall survival was defined as the time from randomization to death from any cause, and progression-free survival was defined as the time from randomization to radiographically confirmed disease progression or death from any cause (whichever occurred first).

Objective response rate is defined as the proportion of patients with a confirmed objective response (complete response or partial response).

Follow-up duration is defined as time from randomization to data cutoff.

Duration of response was defined as the time from first documented evidence of complete or partial response until disease progression or death (whichever occurred first),

**Statistical analyses**

For the Japanese population, an unstratified Cox proportional hazards model with treatment as a covariate was used to estimate unadjusted hazard ratios because of the small sample size. In addition, a multivariate Cox proportional hazards model with treatment, ECOG performance status, HPV status, prior radiation, age, and baseline tumor volume as covariates was used to estimate adjusted hazard ratios as a sensitivity analysis for pembrolizumab-chemotherapy versus EXTREME in the total Japanese subgroup. For the global population, hazard ratios and 95% confidence intervals were estimated using a stratified Cox proportional hazard model with Efron’s method of tie handling.

**Results**

The ITT population for the evaluation of pembrolizumab monotherapy versus EXTREME included all patients randomly assigned to pembrolizumab monotherapy (n = 23) or EXTREME (n = 19). The ITT population for the evaluation of pembrolizumab-chemotherapy versus EXTREME included all 25 patients randomly assigned to pembrolizumab-chemotherapy and the 16 patients assigned to EXTREME while the pembrolizumab-chemotherapy arm was open for enrollment.

**REFERENCE**

[1] Burtness B, Harrington KJ, Greil R, et al. Pembrolizumab alone or with chemotherapy versus cetuximab with chemotherapy for recurrent or metastatic squamous cell carcinoma of the head and neck (KEYNOTE-048): a randomised, open-label, phase 3 study. Lancet. 2019;394:1915–28.

**Supplementary Table 1.** Baseline characteristics of the Japanese subgroup

|  | Pembrolizumab monotherapy  vs EXTREME | | Pembrolizumab with chemotherapy  vs EXTREME | |
| --- | --- | --- | --- | --- |
|  | **Pembrolizumab monotherapy**  ***n* = 23**^a^ | **EXTREME**  ***n* = 19**^a^ | **Pembrolizumab with chemotherapy**  ***n* = 25**^b^ | **EXTREME**  ***n* = 16**^b^ |
| Age, median (range), years | 67 (49-79) | 67 (37-78) | 69 (28-82) | 68 (37-78) |
| Sex |  |  |  |  |
| Male | 21 (91) | 17 (89) | 19 (76) | 14 (88) |
| Female | 2 (9) | 2 (11) | 6 (24) | 2 (13) |
| ECOG performance status |  |  |  |  |
| 0 | 17 (74) | 8 (42) | 12 (48) | 7 (44) |
| 1 | 6 (26) | 11 (58) | 13 (52) | 9 (56) |
| Smoking status |  |  |  |  |
| Current or former | 20 (87) | 18 (95) | 21 (84) | 15 (94) |
| Never | 3 (13) | 1 (5) | 4 (16) | 1 (6) |
| HPV status positive | 3 (13) | 3 (16) | 5 (20) | 3 (19) |
| PD-L1 status |  |  |  |  |
| TPS ≥50% | 6 (26) | 6 (32) | 2 (8) | 6 (38) |
| CPS ≥20 | 14 (61) | 8 (42) | 10 (40) | 7 (44) |
| CPS ≥1 | 21 (91) | 16 (84) | 19 (76) | 14 (88) |
| Disease status^c^ |  |  |  |  |
| Metastatic | 20 (87) | 15 (89) | 16 (64) | 13 (81) |
| Recurrent | 3 (13) | 4 (21) | 8 (32) | 3 (19) |
| Investigator’s choice of platinum for study treatment^d^ |  |  |  |  |
| Carboplatin | 9 (39) | 10 (53) | 14 (56) | 8 (50) |
| Cisplatin | 14 (60) | 9 (47) | 11 (44) | 8 (50) |

CPS, combined positive score; ECOG, Eastern Cooperative Oncology Group; EXTREME, cetuximab plus platinum and 5-fluorouracil; HPV, human papillomavirus; PD-L1, programmed death ligand 1; TPS, tumor proportion score.

^a^The ITT population for the comparison of pembrolizumab monotherapy vs EXTREME included 23 patients randomly assigned to pembrolizumab monotherapy and 19 assigned to EXTREME.

^b^The ITT population for the comparison of pembrolizumab-chemotherapy vs EXTREME included 25 patients randomly assigned to pembrolizumab-chemotherapy and 16 patients assigned to EXTREME while the pembrolizumab-chemotherapy arm was open for enrollment.

^c^One patient in the pembrolizumab + chemotherapy group had neither metastatic disease nor recurrent disease.

^d^Choice of cisplatin or carboplatin was made by the investigator before randomization.

Values are number of patients (%) unless otherwise noted.

**Supplementary Table 2.** Summary of subsequent anticancer therapy for the Japanese subgroup of KEYNOTE-048^a^

| **Anticancer Therapy** | **Pembrolizumab monotherapy**  ***n* = 23** | **Pembrolizumab with**  **chemotherapy**  ***n* = 25** | **EXTREME**  ***n* = 19** |
| --- | --- | --- | --- |
| Any | 16 (70) | 11 (44) | 14 (74) |
| Chemotherapy | 16 (70) | 10 (40) | 8 (42) |
| EGFR inhibitor | 13 (57) | 8 (32) | 2 (11) |
| Kinase inhibitor | 0 | 2 (8) | 0 |
| Immune Checkpoint inhibitor | 2 (9) | 0 | 9 (47) |
| Anti–PD-L1 or Anti–PD-1 | 2 (9)^b^ | 0 | 9 (47) |
| Anti–CTLA-4 | 0 | 0 | 0 |
| Anti–B7-H3 | 0 | 0 | 0 |
| Anti-TIGIT | 0 | 0 | 0 |
| Other immunotherapy | 0 | 0 | 0 |
| Other | 0 | 0 | 0 |

CLTA-4, cytotoxic T-lymphocyte–associated protein 4; EGFR, epidermal growth factor receptor; EXTREME, cetuximab plus platinum and 5-fluorouracil; PD-1, programmed death 1; PD-L1, programmed death ligand 1; TIGIT, T cell immunoreceptor with immunoglobulin and ITIM domains.

^a^All possible subsequent therapies administered after the discontinuation of first-line therapy are included.

^b^Both patients received chemotherapy between study drug discontinuation and initiating a subsequent anti–PD-L1 or anti–PD-1 inhibitor.

**Supplementary Table 3.** Time from randomization to data cutoff (25 February 2019)

|  | Pembrolizumab monotherapy  vs. EXTREME | | Pembrolizumab with chemotherapy  vs. EXTREME | |
| --- | --- | --- | --- | --- |
|  | **Pembrolizumab monotherapy**  ***n* = 23** | **EXTREME**  ***n* = 19** | **Pembrolizumab with chemotherapy**  ***n* = 25** | **EXTREME**  ***n* = 16** |
| Median (range), months | 36.8 (26.3-44.2) | 35.1 (25.3-44.4) | 34.3 (25.7-45.7) | 34.2 (25.3-44.4) |

EXTREME, cetuximab plus platinum and 5-fluorouracil.

Supplementary Table 4. Summary of confirmed objective response by PD-L1 CPS for Japanese patients receiving pembrolizumab monotherapy vs EXTREME

|  | **PD-L1 CPS ≥20** | | **PD-L1 CPS ≥1** | | **Total** | |
| --- | --- | --- | --- | --- | --- | --- |
|  | **Pembrolizumab monotherapy**  ***n* = 14** | **EXTREME**  ***n* = 8** | **Pembrolizumab monotherapy**  ***n* = 21** | **EXTREME**  ***n* = 16** | **Pembrolizumab monotherapy**  ***n* = 23** | **EXTREME**  ***n* = 19** |
| **ORR** | 4 (29) | 1 (13) | 4 (19) | 4 (25) | 4 (17) | 7 (37) |
| **CR** | 1 (7) | 0 | 1 (5) | 1 (6) | 1 (4) | 1 (5) |
| **PR** | 3 (21) | 1 (13) | 3 (14) | 3 (19) | 3 (13) | 6 (32) |
| **SD** | 5 (36) | 5 (63) | 8 (38) | 6 (38) | 9 (39) | 6 (32) |
| **PD** | 5 (36) | 2 (25) | 9 (43) | 6 (38) | 10 (43) | 6 (32) |
| **NN** | 0 | 0 | 0 | 0 | 0 | 0 |
| **NE** | 0 | 0 | 0 | 0 | 0 | 0 |
| **DOR** | 8.4 (3.2 to 36.5+) | 2.6 (2.6-2.6) | 8.4 (3.2 to 36.5+) | 5.5 (2.6 to 31.4+) | 8.4 (3.2 to 36.5+) | 4.1 (2.0 to 31.4+) |

CPS, combined positive score; CR, complete response; DOR, duration of response; EXTREME, cetuximab plus platinum and 5-fluorouracil; NA, no assessment; NE, not evaluable; NN, non-CR and non-PD; ORR, objective response rate (CR + PR); PD, progressive disease; PD-L1, programmed death ligand 1; PR, partial response; SD, stable disease.

Values are number of patients (%) except DOR, which is median (range) months. Database cutoff date: February 25, 2019.

Supplementary Table 5. Summary of confirmed objective response by PD-L1 CPS for Japanese patients receiving pembrolizumab with chemotherapy vs EXTREME

|  | **PD-L1 CPS ≥20** | | **PD-L1 CPS ≥1** | | **Total** | |
| --- | --- | --- | --- | --- | --- | --- |
|  | **Pembrolizumab with chemo**  ***n* = 10** | **EXTREME**  ***n* = 7** | **Pembrolizumab with chemo**  ***n* = 19** | **EXTREME**  ***n* = 14** | **Pembrolizumab with chemo**  ***n* = 25** | **EXTREME**  ***n* = 16** |
| **ORR** | 5 (50) | 1 (14) | 6 (32) | 3 (21) | 8 (32) | 5 (31) |
| **CR** | 1 (10) | 0 | 1 (5) | 1 (7) | 1 (4) | 1 (6) |
| **PR** | 4 (40) | 1 (14) | 5 (26) | 2 (14) | 7 (28) | 4 (25) |
| **SD** | 1 (10) | 5 (71) | 5 (26) | 6 (43) | 7 (28) | 6 (38) |
| **PD** | 4 (40) | 1 (14) | 7 (37) | 5 (36) | 8 (32) | 5 (31) |
| **NN** | 0 | 0 | 0 | 0 | 1 (4.0) | 0 |
| **NE** | 0 | 0 | 1 (5.3) | 0 | 1 (4.0) | 0 |
| **DOR** | 6.9 (5.7 to 26.5+) | 2.6 (2.6-2.6) | 7.5 (5.7 to 26.5+) | 4.1 (2.6 to 31.4+) | 7.5 (4.1 to 26.5+) | 4.1 (2.0 to 31.4+) |

CPS, combined positive score; CR, complete response; DOR, duration of response; EXTREME, cetuximab plus platinum and 5-fluorouracil; NA, no assessment; NE, not evaluable; NN, non-CR and non-PD; ORR, objective response rate (CR + PR); PD, progressive disease; PD-L1, programmed death ligand 1; PR, partial response; SD, stable disease.

Values are number of patients (%) except DOR, which is median (range) months. Database cutoff date: February 25, 2019.

Supplementary Table 6. Summary of treatment-related adverse events that occurred in ≥2 patients in any treatment arm of the Japanese subgroup

|  | **Pembrolizumab monotherapy**  ***n* = 23** | | **Pembrolizumab with chemotherapy**  ***n* = 25** | | **EXTREME**  ***n* = 19** | |
| --- | --- | --- | --- | --- | --- | --- |
|  | **Any grade** | **Grade 3–5** | **Any grade** | **Grade 3–5** | **Any grade** | **Grade 3–5** |
| Any | 17 (74) | 5 (22) | 25 (100) | 19 (76) | 19 (100) | 17 (89) |
| Blood and lymphatic system disorders | 0 | 0 | 14 (56) | 8 (32) | 9 (47) | 7 (37) |
| Anemia | 0 | 0 | 14 (56) | 8 (32) | 8 (42) | 6 (32) |
| Febrile neutropenia | 0 | 0 | 1 (4) | 1 (4) | 3 (16) | 3 (16) |
| Endocrine disorders | 2 (9) | 1 (4) | 4 (16) | 0 | 1 (5) | 0 |
| Hypothyroidism | 1 (4) | 0 | 4 (16) | 0 | 0 | 0 |
| Gastrointestinal disorders | 4 (17) | 0 | 20 (80) | 8 (32) | 18 (95) | 3 (16) |
| Aphthous ulcer | 0 | 0 | 0 | 0 | 2 (11) | 1 (5) |
| Constipation | 1 (4) | 0 | 7 (28) | 0 | 5 (26) | 0 |
| Diarrhea | 1 (4) | 0 | 7 (28) | 0 | 5 (26) | 0 |
| Nausea | 0 | 0 | 19 (76) | 5 (20) | 13 (68) | 1 (5) |
| Stomatitis | 1 (4) | 0 | 10 (40) | 3 (12) | 14 (74) | 1 (5) |
| Vomiting | 1 (4) | 0 | 9 (36) | 1 (4) | 4 (21) | 1 (5) |
| General disorders and administration site conditions | 6 (26) | 1 (4.3) | 18 (72) | 0 | 13 (68) | 0 |
| Face edema | 0 | 0 | 2 (8) | 0 | 0 | 0 |
| Fatigue | 2 (9) | 0 | 3 (12) | 0 | 5 (26) | 0 |
| Malaise | 1 (4) | 0 | 14 (56) | 0 | 6 (32) | 0 |
| Edema | 0 | 0 | 0 | 0 | 2 (11) | 0 |
| Edema peripheral | 0 | 0 | 2 (8) | 0 | 0 | 0 |
| Pyrexia | 3 (13) | 0 | 5 (20) | 0 | 1 (5) | 0 |
| Infections and infestations | 1 (4) | 0 | 8 (32) | 4 (16) | 11 (58) | 0 |
| Lung infection | 0 | 0 | 4 (16) | 2 (8) | 0 | 0 |
| Oral candidiasis | 0 | 0 | 1 (4) | 0 | 2 (11) | 0 |
| Paronychia | 0 | 0 | 0 | 0 | 8 (42) | 0 |
| Injury, poisoning and procedural complications | 2 (9) | 0 | 2 (8) | 0 | 2 (11) | 1 (5) |
| Infusion-related reaction | 1 (4) | 0 | 0 | 0 | 2 (11) | 1 (5) |
| Investigations | 1 (4) | 0 | 22 (88) | 16 (64) | 16 (84) | 11 (58) |
| Alanine aminotransferase increased | 0 | 0 | 2 (8) | 0 | 2 (11) | 1 (5) |
| Aspartate aminotransferase increased | 0 | 0 | 2 (8) | 0 | 3 (16) | 1 (5) |
| Blood creatinine increased | 0 | 0 | 9 (36) | 0 | 1 (5) | 0 |
| Blood magnesium decreased | 0 | 0 | 6 (24) | 0 | 4 (21) | 0 |
| Blood phosphorus decreased | 0 | 0 | 0 | 0 | 1 (5) | 1 (5) |
| Blood potassium decreased | 0 | 0 | 2 (8) | 1 (4) | 3 (16) | 2 (11) |
| Blood potassium increased | 0 | 0 | 4 (16) | 0 | 0 | 0 |
| Blood sodium decreased | 0 | 0 | 4 (16) | 2 (8) | 2 (11) | 1 (5) |
| Lymphocyte count decreased | 0 | 0 | 9 (36) | 7 (28) | 3 (16) | 3 (16) |
| Neutrophil count decreased | 0 | 0 | 18 (72) | 10 (40) | 13 (68) | 11 (58) |
| Platelet count decreased | 0 | 0 | 12 (48) | 2 (8) | 7 (37) | 1 (5) |
| Weight decreased | 0 | 0 | 4 (16) | 0 | 4 (21) | 1 (5) |
| Weight increased | 0 | 0 | 3 (12) | 0 | 1 (5) | 0 |
| White blood cell count decreased | 0 | 0 | 16 (64) | 9 (36) | 13 (68) | 9 (47) |
| Metabolism and nutrition disorders | 2 (9) | 0 | 15 (60) | 6 (24) | 12 (63) | 5 (26) |
| Decreased appetite | 1 (4) | 0 | 14 (56) | 5 (20) | 11 (58) | 3 (16) |
| Hypokalemia |  |  | 2 (8) | 2 (8) | 1 (5) | 1 (5) |
| Hypomagnesemia | 0 | 0 | 1 (4) | 0 | 3 (16) | 1 (5) |
| Nervous system disorders | 0 | 0 | 4 (16) | 2 (8) | 3 (16) | 0 |
| Peripheral sensory neuropathy | 0 | 0 | 2 (8) | 0 | 0 | 0 |
| Respiratory, thoracic and mediastinal disorders | 4 (17) | 0 | 7 (28) | 1 (4) | 6 (32) | 0 |
| Hiccups | 0 | 0 | 6 (24) | 0 | 3 (16) | 0 |
| Oropharyngeal pain | 0 | 0 | 0 | 0 | 2 (11) | 0 |
| Skin and subcutaneous tissue disorders | 13 (57) | 3 (13) | 9 (36) | 0 | 15 (79) | 1 (5) |
| Alopecia | 0 | 0 | 6 (24) | 0 | 2 (11) | 0 |
| Dermatitis acneiform | 3 (13) | 0 | 0 | 0 | 8 (42) | 0 |
| Dry skin | 0 | 0 | 1 (4) | 0 | 5 (26) | 1 (5) |
| Pruritus | 5 (22) | 0 | 1 (4) | 0 | 2 (11) | 0 |
| Rash | 6 (26) | 1 (4) | 1 (4) | 0 | 4 (21) | 0 |

Data are n (%).

EXTREME, cetuximab plus platinum and 5-fluorouracil.

Supplementary Figure 1. Trial population for the Japanese subgroup of KEYNOTE-048


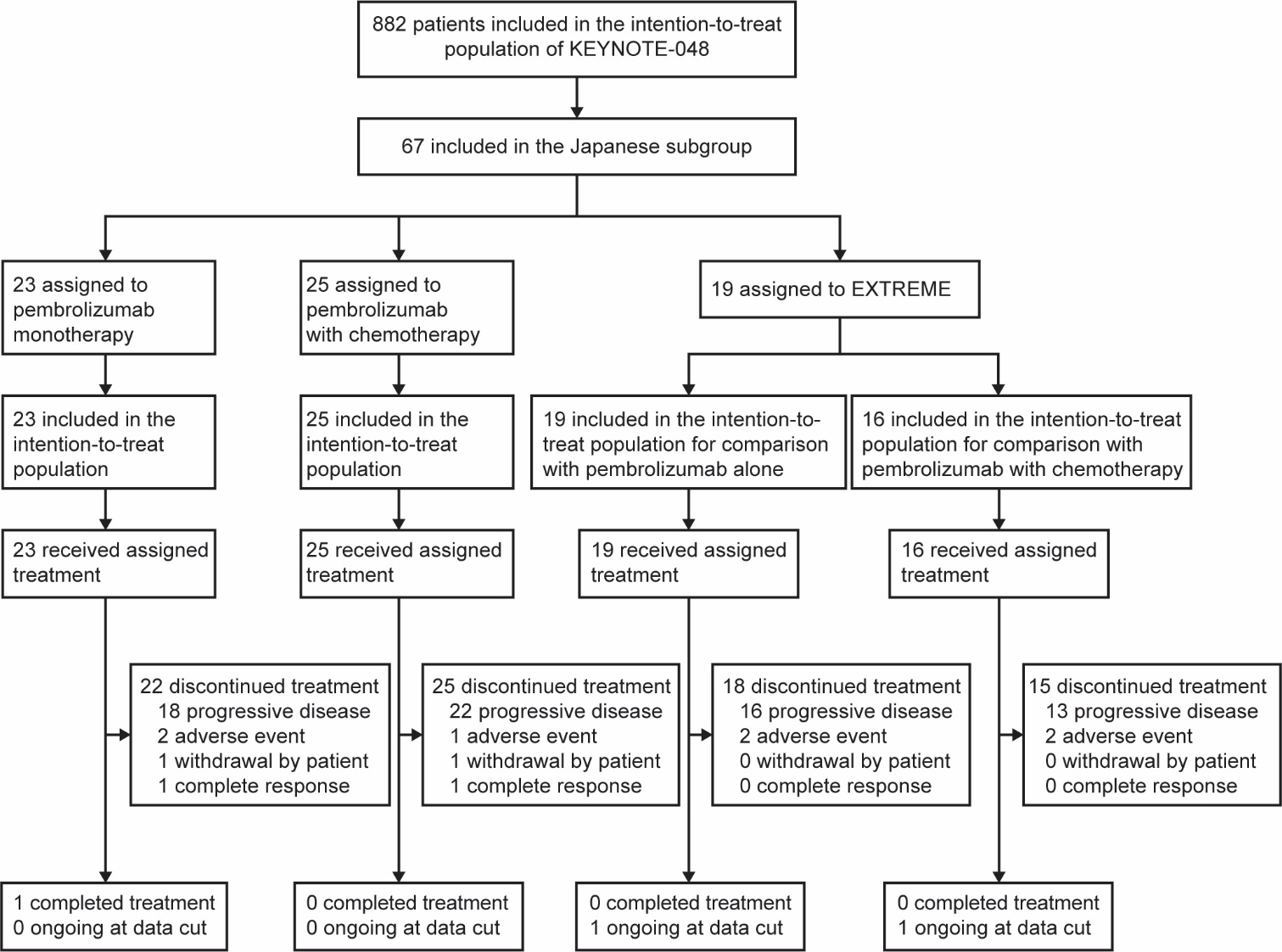

Supplement: Supplementary file 1 — Supplementary file1 (DOCX 390 KB) Methods: study design and patients, procedures, outcomes, statistical analyses. Table S1. Baseline characteristics of the Japanese subgroup. Table S2. Summary of subsequent anticancer therapy for the Japanese subgroup of KEYNOTE-048. Table S3. Time from randomization to data cutoff (25 February 2019). Table S4. Summary of confirmed objective response by PD-L1 CPS for Japanese patients receiving pembrolizumab monotherapy vs EXTREME. Table S5. Summary of confirmed objective response by PD-L1 CPS for Japanese patients receiving pembrolizumab with chemotherapy vs EXTREME. Table S6. Summary of treatment-related adverse events that occurred in ≥ 2 patients in any treatment arm of the Japanese subgroup. Figure S1. Trial population for the Japanese subgroup of KEYNOTE-048 [file 10147_2022_2233_MOESM1_ESM.docx]
